# Circular RNAs and immunotherapy in retinoblastoma: emerging biomarkers and precision therapeutic strategies

**DOI:** 10.3389/fimmu.2025.1666606

**Published:** 2025-09-17

**Authors:** Xueting Wang, Jiakang Ma, Yalong Dang, Fang Lei

**Affiliations:** ^1^ School of Information Engineering, Henan University of Science and Technology, Luoyang, China; ^2^ Department of Ophthalmology, The First Affiliated Hospital, and College of Clinical Medicine of Henan University of Science and Technology, Luoyang, China; ^3^ Henan Key Laboratory of Cancer Epigenetic, Cancer Institute, The First Affiliated Hospital, and College of Clinical Medicine of Henan University of Science and Technology, Luoyang, China; ^4^ Department of Ophthalmology, Sanmenxia Central Hospital/Affiliated Sanmenxia Central Hospital of Henan University of Science and Technology, Sanmenxia Eye Hospital, International Joint Laboratory for Glaucoma Aqueous Humor Drainage in Henan Province, Engineering Technology Research Center for Minimally Invasive Eye Surgery in Henan Province, and Engineering Research Center for Minimally Invasive Ophthalmic Surgery in Henan Province, Sanmenxia, China

**Keywords:** retinoblastoma, circular RNAs, tumor metastasis, drug resistance, biomarkers, immunotherapy

## Abstract

Retinoblastoma (RB) immunotherapy represents a paradigm shift in managing this aggressive pediatric eye cancer, overcoming limitations of conventional therapies. Recent breakthroughs reveal how circular RNAs (circRNAs) critically modulate the tumor-immune microenvironment: oncogenic circRNAs promote immune evasion by upregulating PD-L1 and suppressing T cell activity, while tumor-suppressive circRNAs such as circMKLN1 enhance antigen presentation and cytotoxic responses. The convergence of circRNA biology with immunotherapy has yielded innovative strategies, including circRNA-targeted immune checkpoint blockade to reverse T cell exhaustion, circRNA-engineered CAR-T cells with improved tumor homing and persistence, and circRNA-based oncolytic viruses that stimulate immunogenic cell death. Notably, exosomal circRNAs serve dual roles as both immune modulators and minimally invasive biomarkers for predicting immunotherapy response. While preclinical studies demonstrate remarkable synergy between circRNA inhibition and PD-1/CTLA-4 blockade in RB models, clinical translation requires optimization of delivery systems and combinatorial regimens. This review summarizes the latest evidence positioning circRNAs as central regulators of anti-tumor immunity and provides a strategic roadmap for the integration of circRNA-based approaches in precision immunotherapy for RB.

## 1 Introduction

Retinoblastoma (RB) is the most prevalent intraocular malignancy in children (1). It poses a severe threat to both vision and survival in infants and young children. Current therapeutic strategies, including chemotherapy, enucleation, laser photocoagulation, and cryotherapy, have improved outcomes but remain limited by non-specific toxicity, treatment resistance, and metastatic relapse (2, 3). To address these limitations, immunotherapy has emerged as a transformative approach in RB treatment, capable of enhancing tumor specificity, reducing systemic toxicity, and preventing recurrence through durable immune activation (3). Recent studies highlight the critical role of immune evasion in RB progression, with tumors often overexpressing PD-L1 and exhibiting T cell exhaustion signatures (4, 5). These findings have spurred interest in checkpoint inhibitors, CAR-T cells, and other immunomodulatory strategies tailored to RB (6).

In parallel, circular RNAs (circRNAs), a class of covalently closed-loop non-coding RNAs, have gained attention for their regulatory roles in tumor biology, including epigenetic control, cell proliferation, metastasis, and chemoresistance (7, 8). Notably, circRNAs influence immune responses by modulating antigen presentation, cytokine signaling, and immune checkpoint expression (9, 10). However, the immunological roles of circRNAs in RB remain underexplored. This review provides a comprehensive summary of the recent advances in circRNA research and immunotherapeutic strategies in the context of retinoblastoma, with the aim of highlighting novel insights into molecular mechanisms and translational application.

## 2 The regulatory role of circRNAs in RB progression

### 2.1 Overview of circular RNAs

Circular RNAs (circRNAs) are covalently closed-loop, non-coding RNAs that are widely expressed across diverse organisms. They primarily arise through back-splicing of precursor transcripts (11). This circular structure renders them resistant to exonuclease degradation, ensuring remarkable stability and prolonged half-lives, with approximately 80% localized in the cytoplasm (12). CircRNAs can be classified into intronic, exonic (ecircRNAs), and exon-intron forms, with ecircRNAs being the most prevalent (13, 14). Functionally, circRNAs act as microRNA (miRNA) sponges, sequestering miRNAs via the competing endogenous RNA (ceRNA) mechanism to modulate mRNA expression (15). Additionally, they interact with RNA-binding proteins, regulating RNA stability, transcription, and splicing, while also forming complexes with other RNAs that influence epigenetic and transcriptional processes (16, 17). Recent findings suggest that certain circRNAs possess coding potential, producing functional peptides (18). CircRNAs are increasingly recognized as pivotal regulators in tumorigenesis. For instance, circZNF566 promotes hepatocellular carcinoma progression by sponging miR-4738-3p, while circ-0052112 enhances breast cancer metastasis through targeting miR-125a-5p (19, 20). In RB, circRNAs play significant roles in oncogenesis, diagnosis, therapy response, and prognosis, positioning them as promising biomarkers and therapeutic targets (21).

### 2.2 Oncogenic circRNAs

#### 2.2.1 circ-0000527 and circ-0000034

circ-0000527, also known as circ-FAM158A, is the sole circular RNA transcribed from the FAM158A gene and is notably overexpressed in RB (22). Functional investigations have demonstrated that silencing circ-0000527 inhibits RB cell proliferation, migration, invasion, angiogenesis, and induces apoptosis (22–24). Mechanistically, circ-0000527 functions as a molecular sponge for miR-646, leading to the upregulation of LRP6 and BCL-2. These molecules promote tumor progression via Wnt/β-catenin signaling and inhibit apoptosis, respectively (25, 26). Furthermore, circ-0000527 regulates the miR-27a-3p/HDAC axis, decreasing PI3K and AKT phosphorylation, which enhances epithelial–mesenchymal transition (EMT), drug resistance, and angiogenesis (27). Besides, circ-0000527 reduces caspase activity through the miR-98-5p/XIAP axis, further suppressing apoptosis (22). As a competing endogenous RNA (ceRNA) for miR-138-5p, it upregulates SLC7A5, facilitating amino acid uptake to satisfy the metabolic demands of tumor cells (24). These diverse mechanisms underscore circ-0000527 as a key oncogenic regulator in RB.

circ-0000034, also referred to as circ-001787 or circDHDDS, is derived from the DHDDS gene, mutations in which are linked to retinitis pigmentosa (28, 29). circ-0000034 has been shown to enhance RB cell viability, migration, invasion, autophagy, and EMT. Knockdown of circ-0000034 results in G0/G1 cell cycle arrest and induces apoptosis in RB cells (30, 31). To date, miR-361-3p is the only confirmed target of circ-0000034 in RB, acting as a tumor suppressor (32). As a ceRNA for miR-361-3p, circ-0000034 modulates downstream targets, including WNT3A and STX17 (31). By sponging miR-361-3p, circ-0000034 upregulates WNT3A, thereby promoting oncogenesis (29). Additionally, ADAM19, a disintegrin and metalloprotease involved in cell adhesion and proteolysis, is a downstream target of miR-361-3p, enhancing RB cell migration and invasiveness (28).

#### 2.2.2 circ-0075804

circ-0075804, a circular RNA originating from the E2F3 gene located on chromosome 6, plays a pivotal role in the regulation of cell cycle-associated genes through its direct interaction with the RB protein (33). E2F3, as a transcription factor, regulates critical genes involved in the cell cycle and cellular proliferation, and its activity is tightly controlled by RB binding. In RB tissues and cell lines, an upregulation of circ-0075804 has been shown to promote cellular proliferation and malignant transformation, while simultaneously inhibiting apoptosis, a hallmark of cancer progression (34, 35). This effect is thought to be mediated by circ-0075804’s interaction with heterogeneous nuclear ribonucleoproteins, which stabilizes E2F3 mRNA, thereby enhancing its activity and contributing to oncogenic processes (35). ROCK1, a serine/threonine kinase of the AGC family encoded at 18q11.1, regulates various cellular processes, including proliferation, differentiation, adhesion, and cytoskeletal remodeling (36). circ-0075804 activates the ROCK1 pathway via miR-204-5p inhibition, thereby contributing to RB progression (36). Additionally, circ-0075804 serves as a sponge for miR-138-5p, which modulates the 3’-untranslated region (UTR) of the PEG10 gene. This interaction promotes tumor growth, underscoring its involvement in RB pathogenesis (37). Notably, circ-0075804 also targets miR-1287-5p, thereby upregulating LIMS1, a cytoskeletal scaffold protein that enhances cellular invasiveness and contributes to RB metastasis (38). These findings highlight circ-0075804 as a multifaceted regulator, influencing several key pathways in the progression and metastasis of RB.

#### 2.2.3 Other oncogenic circRNAs

In addition to the aforementioned circRNAs, several others have emerged as significant players in the pathogenesis of RB. These include circRNF20, circ-ODC1, circ-0099198, circROBO1, and circ-0000989, all of which have been implicated in RB tumorigenesis (39, 40). PAX6 is indispensable for retinal and ocular development; its dysregulation has been linked to congenital aniridia, anterior segment dysgenesis, glaucoma, and RB (41). Notably, circRNF20 has been shown to promote RB progression by directly targeting the tumor-suppressive miR-132-3p, thereby indirectly upregulating PAX6 expression, which further contributes to tumorigenesis (39). Moreover, both the long non-coding RNA ZFPM2-AS1 and miR-130a-3p are involved in regulating RB development and chemoresistance, with a particular focus on their interaction with PAX6 (42, 43). The role of circ-ODC1 in RB progression is also of particular interest, as it encodes ODC1, a rate-limiting enzyme in polyamine biosynthesis, which has been recognized as an oncogene in a range of malignancies (44). Du et al. demonstrated that circ-ODC1 fosters RB progression by sponging miR-422a, resulting in the activation of SKP2 (40). SKP2 participates in ubiquitination, autophagy, cell cycle regulation, and signal transduction. Inhibiting SKP2 sensitizes non-small cell lung cancer and osteosarcoma cells to cisplatin both *in vitro* and *in vivo*, potentially overcoming drug resistance (45). Furthermore, circ-0099198 promotes the proliferation and metastasis of Y79 and SO-RB50 cells by regulating the miR-1287/LRP6 axis, accelerating cell cycle progression (46).

### 2.3 Tumor-suppressive circRNAs

#### 2.3.1 circMKLN1

circMKLN1, a circular RNA derived from the MKLN1 gene, plays a critical role in the autophagic processes underlying diabetic retinopathy, where it has been shown to promote neovascularization (47). MKLN1 encodes a muscle protein that is pivotal in cell proliferation by mediating cytoskeletal organization and cellular motility (48). Notably, the upregulation of circMKLN1 facilitates its interaction with miR-425-5p, thereby regulating the expression of PDCD4, a key tumor suppressor and a downstream target of RB signaling (49). circMKLN1 promoted CDK8 expression through sponge adsorption of miR-26a/b, which regulates EMT (50). Notably, silencing of PDCD4 markedly attenuates the tumor-suppressive effects of circMKLN1 (49). Beyond these effects, PDCD4 constrains AP-1–dependent transcription and cap-dependent translation. Thus, the circMKLN1–miR-425-5p–PDCD4 axis plausibly couples transcriptional restraint (c-MYC programs) with suppression of invasion (MMP9, vimentin) and maintenance of epithelial identity (E-cadherin) (49, 51). Functionally, this positions circMKLN1 to counter EMT and metastatic competence while limiting proliferative signaling, providing a mechanistic counterweight to oncogenic circRNAs that amplify Wnt/β-catenin or PI3K/AKT activity (52).

#### 2.3.2 circ-0001649

circ-0001649, a transcriptional product derived from the DNA repair gene SHPRH, as a tumor suppressor by negatively regulating tumorigenesis via the Wnt/β-catenin signaling pathway (53). This circRNA has been linked to several malignancies, including prostate, gastric, and hepatic cancers (54). circ-0001649 exerts its antitumor effects by inhibiting the AKT/mTOR pathway—a pivotal signaling axis that governs cellular processes such as proliferation, apoptosis, angiogenesis, and glucose metabolism in cancer cells. Both *in vitro* and *in vivo* studies have shown that overexpression of circ-0001649 results in reduced expression of AKT and mTOR, accompanied by diminished RB cell viability, inhibition of proliferation, and promotion of apoptosis (55). Furthermore, the PI3K/AKT/mTOR signaling pathway promotes forward regulation that stabilizes β-catenin and facilitates EMT. Inhibition of this pathway by circ-0001649 thus provides dual antitumor effects: attenuating anabolic growth processes while simultaneously suppressing β-catenin-mediated transcriptional activity (56, 57). In RB, where cell-cycle regulation intersects at the RB/E2F node, attenuation of the AKT/mTOR pathway by circ-0001649 is proposed to diminish cyclin-CDK activity, thereby reinforcing G1-phase arrest and sensitizing tumor cells to cytotoxic stress (58).

#### 2.3.3 circ-0093996 and circCUL2

circ-0093996 is derived from the TET1 gene which functions as a pivotal tumor suppressor gene (59). The overexpression of circ-0093996 induces G0/G1 cell cycle arrest in Y79 and WERI-RB1 cells, effectively halting the progression of the cell cycle. This regulatory effect is mediated through the sequestration of miR-492 and miR-494-3p, which leads to the downregulation of key oncogenes, including β-catenin and c-MYC, while simultaneously upregulating GSK-3 (60, 61). GSK-3, a highly conserved serine/threonine kinase, inhibits β-catenin activity and prevents its cytoplasmic accumulation, thereby suppressing Wnt/β-catenin signaling. Ultimately, this cascade inhibits the proliferation, migration, and invasiveness of RB cells (61). circCUL2 arises from the back-splicing of CUL2 mRNA, which encodes an E3 ubiquitin ligase, and is involved in cell cycle regulation (62). E2F2, a member of the E2F transcription factor family, is an RB-regulated transcription factor that governs cell invasion and proliferation during cancer progression and erythroid maturation. circCUL2 acts as a ceRNA for miR-214-5p, thereby modulating the expression of E2F2 (62). In RB cells, circCUL2 is upregulated and indirectly represses E2F2 expression, resulting in inhibition of tumor angiogenesis and attenuation of RB cell proliferation and migration (63).

### 2.4 circRNAs in RB drug resistance

Drug resistance in RB involves complex molecular mechanisms, including circRNA-mediated regulation of oncogenic pathways. circ-0000527, circ-0000034, and circ-0093996 promote chemoresistance via the Wnt/β-catenin axis, while circODC1 and circRNF20 suppress resistance by targeting SKP2 and PAX6, respectively. *In vivo*, silencing circ-0000034 reduced RB tumor volume and weight, likely through downregulation of stemness markers CD133 and SOX2 (29). Mechanistically, circ-0000034 enhances autophagy by binding STX17, facilitating autophagosome–lysosome fusion. Depletion of circ-0000034 in Y79 and WERI-Rb1 cells leads to a marked reduction in the expression of LC3-II/LC3-I and Beclin-1, which impairs autophagic flux and diminishes drug resistance (31, 47). These findings suggest that circ-0000034–induced autophagy contributes to RB chemoresistance (64). Therefore, strategies targeting circRNAs may present a promising therapeutic approach to overcoming drug resistance and improving the prognosis of RB patients.

## 3 Prospects of circRNAs in the diagnosis and treatment of RB

### 3.1 circRNAs as potential diagnostic biomarkers for RB

Current clinical diagnosis of RB relies heavily on imaging techniques such as MRI, CT, and B-scan ultrasonography (65). However, the intraocular location of RB hinders tissue biopsy, and the young age of patients limits diagnostic compliance and accuracy (66). To overcome these challenges, non-invasive strategies like liquid biopsies based on molecular biomarkers have gained interest (67). circRNAs, characterized by high stability and tissue specificity, have emerged as promising diagnostic candidates. Lyu et al. (68) identified 550 downregulated circRNAs in RB tissues via RNA sequencing, with circ-0093996 most significantly reduced. Additional studies have reported differential expression of circRNAs such as circ-0000034, circ-0075804, circ-0000527, circ-0000989, and circ-0001649 in RB tumor tissues (34, 55, 69), suggesting their diagnostic potential. Nonetheless, most studies have focused on tumor samples and primary cell lines, limiting clinical translation. Recently, exosomal circRNAs have attracted attention due to their stability and presence in biofluids, enabling minimally invasive detection and real-time disease monitoring (70, 71). An et al. (39) demonstrated that serum-derived exosomes from RB patients contained elevated levels of circRNF20, especially in advanced TNM stages. Functionally, these exosomes enhanced viability and proliferation of RB cell lines, indicating that exosomal circRNF20 may contribute to RB progression and serve as a non-invasive biomarker.

### 3.2 circRNAs as therapeutic targets and prognostic biomarkers in RB

**Figure 1:**
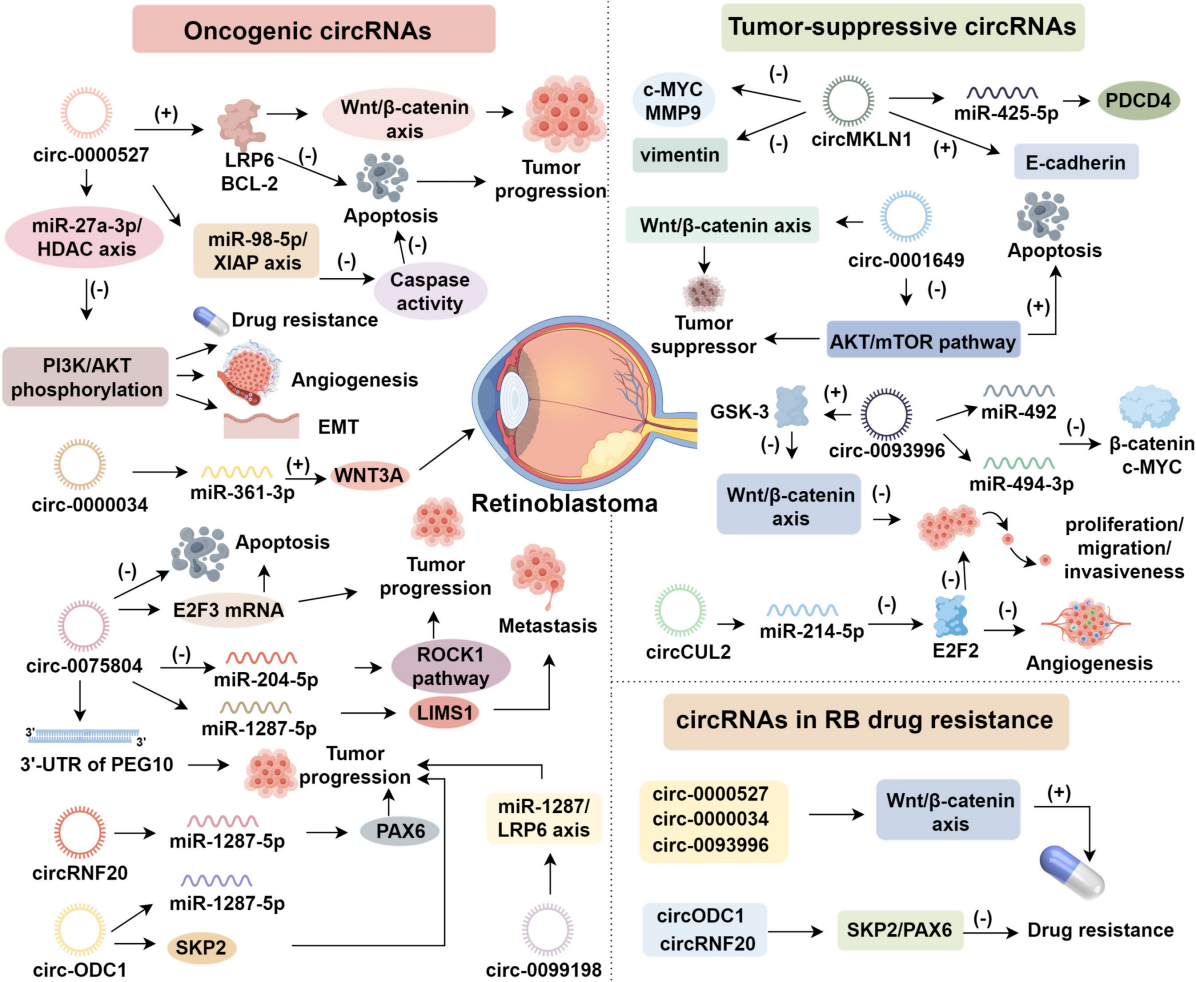
The regulatory role of circRNAs in retinoblastoma progression.

circRNAs have emerged as potential biomarkers in RB (72). Elevated levels of oncogenic circRNAs—such as circ-0119412, circRNF20, and circ-0000034—are associated with higher TNM stage, optic nerve invasion, and poor survival outcomes (28, 73). In contrast, reduced expression of tumor-suppressive circRNAs such as circ-0001649 predicts more aggressive disease, whereas circMKLN1 overexpression correlates with favorable prognosis (74, 75). Functional studies highlight the therapeutic relevance of circRNAs. Knockdown of circRNF20 via shRNA in RB cells reduced proliferation and colony formation by 50%, and xenograft models confirmed tumor volume reductions of similar magnitude (39). Likewise, circMKLN1 overexpression in Y79 and WERI-RB1 cells suppressed colony formation by over 60% and significantly reduced tumor burden *in vivo* (49). Additional circRNAs, including circ-0000034, circ-0075804, circ-ODC1, circ-0099198, and circ-0007534, exert growth-suppressive effects upon silencing, while overexpression of circ-0093996 yields similar outcomes. CircRNAs may also influence drug response. For instance, circ-0007534 overexpression diminishes the antitumor efficacy of osthole, reversing its inhibitory effects on cell viability and colony formation (76). Collectively, these findings underscore the dual role of circRNAs as both biomarkers and therapeutic targets, offering promising avenues to overcome chemoresistance and improve RB treatment outcomes (**Figure 1**).

## 4 Advances in immunotherapy

Recent advances in immunotherapy have transformed the therapeutic landscape of RB, offering strategies that harness and redirect immune responses against tumor cells. Immune checkpoint inhibitors (ICIs), such as PD-1, PD-L1, and CTLA-4 blockers, restore cytotoxic T cell–mediated surveillance by disrupting inhibitory signals within the tumor microenvironment (TME), and have shown promising efficacy in RB, where PD-L1 is upregulated and co-expression of PD-1/CTLA-4 correlates with poor prognosis (77, 78). Adoptive cell therapy (ACT), including tumor-infiltrating lymphocyte (TIL) and CAR-T approaches, enables personalized administration of antitumor lymphocytes (79). CAR-T cells targeting CD171 and GD2 effectively eliminate primary and metastatic RB cells and mitigate chemotherapy-associated toxicity (80, 81). Parallelly, inhibitors of cyclin-dependent kinases 4/6 (CDK4/6) such as palbociclib, ribociclib, and abemaciclib suppress Rb phosphorylation and cell cycle progression, while concurrently enhancing antitumor immunity by increasing antigen presentation and suppressing regulatory T cell (Treg) proliferation (82, 83). TFF1-overexpressing RB cells exhibit CDK6 downregulation, and exogenous TFF1 impairs the viability of RB cell lines, supporting the clinical utility of CDK4/6 inhibitors (84, 85). Anti-angiogenic therapy targeting VEGF, which is overexpressed in poorly differentiated RB, limits neovascularization and immunosuppression in the TME. Bevacizumab, an anti-VEGF monoclonal antibody, has shown efficacy in RB xenograft models, further validating VEGF as a viable target (86). Oncolytic viruses (OVs), particularly engineered adenoviruses, selectively replicate within and lyse tumor cells while stimulating systemic immune responses. Intra-tumoral delivery enhances local efficacy and minimizes systemic toxicity (87). Mouse models of RB treated with replication-competent and -deficient adenoviruses have demonstrated potent antitumor effects and synergism with chemo- or radiotherapy (88). Moreover, therapeutic cancer vaccines, especially DNA-based platforms, reawaken suppressed immunity by expanding tumor-reactive T cell pools. DNA vaccines offer multivalent, HLA-independent responses, favorable safety, and scalability (89). Racotumomab, an anti-idiotype vaccine targeting NeuGcGM3, has elicited strong immune responses in RB patients in clinical trials (90, 91).

Recent studies have begun to elucidate how circRNAs shape the immunological landscape of RB. Oncogenic circRNAs such as circ-0136666 and circ-0000512 promote immune escape by upregulating PD-L1 expression, either directly or via modulation of Wnt/β-catenin and ROCK1 pathways, which are known to suppress antigen presentation and impair CD8^+^ T cell cytotoxicity (92, 93). Conversely, tumor-suppressive circRNAs like circMKLN1 enhance anti-tumor immunity by upregulating PDCD4, a key regulator of antigen processing and presentation, thereby facilitating CD8^+^ T cell activation (49). Furthermore, circMKLN1 overexpression has been shown to elevate E-cadherin levels and reduce c-MYC and MMP9 expression, collectively fostering a TME more conducive to T cell infiltration and activation (52). Exosomal circRNAs such as circ-SPEF2 may also influence immune dynamics by promoting the expansion of immunosuppressive cell subsets, including Tregs (94), though direct evidence in RB remains limited. These findings highlight the dual roles of circRNAs as both intrinsic regulators of immune checkpoints and extrinsic communicators via exosomal signaling, underscoring their potential as targets for immunotherapy (95). Compared to conventional therapies, immunotherapy provides superior specificity, minimizes off-target toxicity, and potentially penetrates the blood–retinal and blood–brain barriers (96, 97). As the immunobiology of RB becomes clearer, and immune modulation strategies such as checkpoint blockade, ACT, CDK4/6 inhibition, anti-VEGF therapy, oncolytic virotherapy, and vaccination continue to evolve, these immunotherapeutic advances offer a compelling framework for improved RB management and expanded treatment paradigms (98).

## 5 Conclusion

Retinoblastoma therapy has entered a transformative era, driven by advances in circRNA biology and immunotherapeutic innovation. Circular RNAs (circRNAs), owing to their stability and tissue specificity, have emerged as critical regulators of tumorigenesis, chemoresistance, and metastatic progression. Moreover, their potential for non-invasive diagnostics, particularly through exosomal detection (circRNF20), offers new opportunities for early detection and monitoring. In parallel, immunotherapies including checkpoint inhibitors, CAR-T cells, and oncolytic viruses are overcoming the limitations of traditional therapies by harnessing immune-mediated tumor clearance and facilitating durable remission.

Despite these exciting developments, several knowledge gaps remain before clinical translation. Key challenges include optimizing *in vivo* delivery systems for circRNA-targeted therapeutics, identifying tumor-specific circRNA targets with minimal off-target effects, integrating circRNA modulation with immunotherapies to enhance efficacy and reduce resistance, and establishing exosomal circRNAs as biomarkers for treatment stratification and disease monitoring. Clinical validation requires robust preclinical RB models that mimic the human tumor microenvironment and prospective trials to assess safety, specificity, and long-term outcomes. The synergy between circRNA modulation and immune activation offers a strategy to overcome drug resistance and metastatic relapse. As the molecular landscape of RB is further understood, integrating circRNA diagnostics with precision immunotherapy promises personalized, low-toxicity therapies. Future efforts should focus on bridging preclinical findings to clinical applications, improving survival and quality of life for pediatric RB patients.
